# Two cases of spontaneous cervical epidural hematoma without back or neck pain in elderly Japanese men

**DOI:** 10.1002/ams2.317

**Published:** 2017-10-24

**Authors:** Takashi Hongo, Kenichi Iseda, Midori Tsuchiya, Mototaka Inaba, Satoshi Nozaki, Kenji Takahashi, Masaaki Nakajima, Toshifumi Fujiwara

**Affiliations:** ^1^ Emergency Department Okayama Saiseikai General Hospital Okayama Japan; ^2^ Department of Neurosurgery Okayama Saiseikai General Hospital Okayama Japan

**Keywords:** Back pain, corpectomy, dysstasia, elderly, spontaneous spinal epidural hematoma

## Abstract

**Cases:**

Spontaneous spinal epidural hematoma (SSEH) is an uncommon disease. Most SSEH cases involve back and/or neck pain. We report the cases of two men who experienced SSEH with dysstasia but without back or neck pain.

**Outcomes:**

This study presents two cases involving elderly Japanese men who visited an emergency department because of sudden dysstasia without back or neck pain. The results of the neurological examinations revealed ataxic gait. Cervical spinal epidural hematomas were observed by computed tomography and magnetic resonance imaging. One patient underwent hematoma removal and decompression by corpectomy, whereas the other patient received conservative treatment and observation. The patients were discharged without sequelae.

**Conclusion:**

Spinal epidural hematomas are difficult to diagnose, and a delayed diagnosis can adversely affect the patient's quality of life. These hematomas should be considered in the differential diagnosis of cerebrovascular diseases.

## Background

Spontaneous spinal epidural hematoma (SSEH) is an uncommon disease that is characterized by an acute onset of quadriplegia or paraplegia. Previous case reports have indicated that almost 90% of SSEH cases involve back and/or neck pain.^1^ We report the cases of two elderly Japanese men who experienced spontaneous cervical epidural hematomas with dysstasia but without back or neck pain.

## Cases

### Case 1

**Figure 1:**
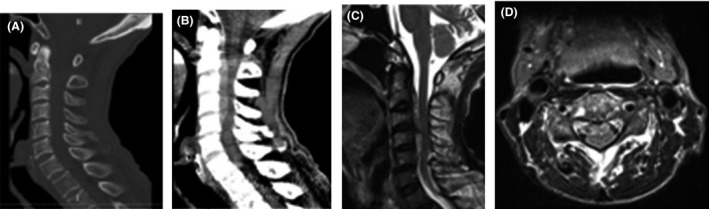
Imaging findings from case 1, an 82‐year‐old Japanese man who suddenly developed dysstasia without back or neck pain. A, B, Bone‐specific computed tomography reveals no definite findings, although a high‐density soft tissue mass is visible in the spinal canal at C2–C5. C, D, Sagittal and axial T2‐weighted magnetic resonance imaging reveals a spontaneous spinal epidural hematoma on the ventral side of the spinal cord at C2–C5.

**Figure 2:**
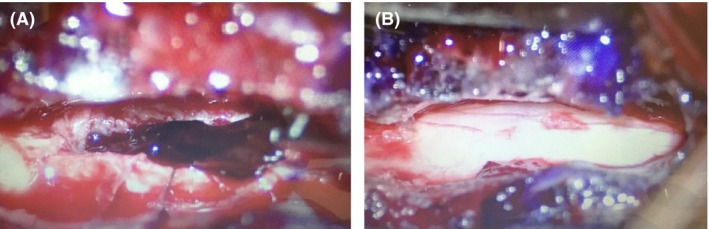
Oblique corpectomy at C3–C5 was undertaken to remove the epidural hematoma from an 82‐year‐old Japanese man who suddenly developed dysstasia (case 1).

An 82‐year‐old Japanese man suddenly developed dysstasia without back or neck pain, which made him unable to walk and barely able to stand. He was previously able to perform moving exercises of his neck every morning. Thus, he was admitted to our hospital. He had a medical history of cerebral infarction (treated using 100 mg/day aspirin), dementia, and hypertension but had no history of head and neck trauma. The patient was alert on admission, and his blood pressure was 163/57 mmHg. Neurological examination revealed the presence of astasia–abasia. The Romberg test was also positive. However, the rest of his physical examinations, such as the test for motor or sensory deficits, had unremarkable findings. Moreover, the patient had no coagulation disorder, based on blood test results. The cervical bone conditional computed tomography (CT) revealed no specific findings, although the soft tissue conditional CT showed a high‐density mass at C2–C5. Furthermore, magnetic resonance imaging (MRI) of the cervical spine displayed a ventral extradural lesion at C2–C5, which was iso‐/hyperintense and hyperintense to the cord on T1‐ and T2‐weighted images, respectively (Fig. 1). Based on these findings, we diagnosed the patient with SSEH, which extended from C2 to C5. Considering that his symptoms did not improve after 24 h, we performed hematoma removal and decompression by corpectomy from the front of C3–C5 at approximately 32 h after the onset of his symptoms (Fig. 2). The surgery time was 4.4 h. Subsequently, he was able to stand and walk 3 days after the operation without fixation. Currently, the patient can walk without assistance and has not experienced recurrence for 1 year.

### Case 2

**Figure 3:**
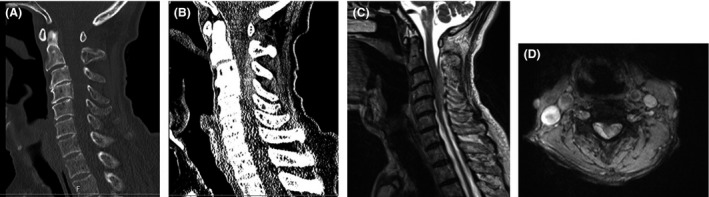
Imaging findings from case 2, a 90‐year‐old Japanese man suddenly developed dysstasia without back or neck pain. A, B, Bone‐specific computed tomography reveals no definite findings, although a high‐density soft tissue mass is visible in the spinal canal at C2–C5. C, D, Sagittal and axial T2‐weighted magnetic resonance imaging reveals a spontaneous spinal subdural hematoma on the dorsal side of the spinal cord at C3–C5.

A 90‐year‐old Japanese man suddenly developed dysstasia without back or neck pain and was transferred to our hospital. He had a medical history of hypertension and dementia but had no history of trauma or previous antiplatelet medication use. The patient was alert on admission, with a blood pressure of 154/93 mmHg. Neurological examination revealed the presence of astasia–abasia. The Romberg test was also positive. The rest of his physical examinations, such as the test for motor or sensory deficits, showed unremarkable findings. Furthermore, the patient had no coagulation disorder, based on blood test results. The head MRI revealed no specific findings, although the soft tissue conditional CT displayed a high‐density mass at C2–C5. In addition, the MRI of the cervical spine showed a dorsal extradural lesion at C2–C5, which was isointense and hyperintense to the cord on T1‐ and T2‐weighted images, respectively (Fig. 3). The cervical spinal cord was normal. Based on these findings, we diagnosed the patient with SSEH, which extended from C2 to C5. We started conservative treatment using methylprednisolone (500 mg/day), tranexamic acid, and mecobalamin. The patient's symptoms gradually improved after 24 h without further worsening. We decided to manage the patient using conservative treatment because of the observed improvements in his neurological deficits and advanced age (>90 years). He was discharged on day 19 without any neurological problems.

## Discussion

A SPINAL EPIDURAL hematoma is a rare but disabling disease, and SSEH is frequently described as a hematoma occurring in the absence of trauma or iatrogenic procedure. The cause of spinal epidural hematoma in 40–50% of cases is not known.^2^ The causes of SSEH, such as increased bleeding tendency with the use of various medications,^3^ blood dyscrasia,^4^ direct or indirect spinal trauma,^5^ and hypertension,^6^ have been discussed in published works. However, the mechanism of spinal epidural hematoma is not clear. Most researchers have asserted that spinal epidural hematoma arises from epidural venous plexus in the spinal space.^7^ The fluctuations in intrathoracic and intra‐abdominal pressures after exercise or other maneuvers induce rupture of a delicate vein in the valveless epidural plexus.^7^ In case 1, no specific source of bleeding, such as vascular malformation or tumor, was observed during surgery. The increased venous pressure caused by cervical movement, high blood pressure, and antiplatelet medication induced hematoma. In case 2, the only risk factor for SSEH was high blood pressure. The locations of SSEH appear to have a bimodal distribution with peaks observed at C6 and T12.^8^ These SSEH lesions are usually located dorsal to the spinal cord.^2^ Spontaneous spinal epidural hematoma generally develop in individuals between the ages of 50 and 80 years.^9^

Most patients with SSEH present with severe back and/or neck pain, which often involves a radicular component.^10^ The pain is usually followed by progressive motor or sensory deficits. Liu *et al*. reported that 2 of 23 SSEH cases did not involve back and/or neck pain.^1^ For these cases, the establishment of a diagnosis is difficult. The unusual feature of our cases was that our patients only presented with sudden‐onset dysstasia without pain, and they did not have any cerebellar or brain stem lesions. In our cases, both patients were >80 years old and had dementia. These factors might obscure back or neck pain because they are unable to communicate their discomforts given their declining brain function and abilities. In these circumstances, pain among the elderly often becomes unrecognized. The physical findings, such as positive Romberg test and sudden‐onset dysstasia, were suspected to be associated with the presence of posterior column syndrome and vascular disease.

No definitive strategy for the treatment of SSEH has been established, although the most common approach is early surgical intervention. Groen *et al*.^8^ reported that early surgical interventions are associated with better neurological and functional recovery. However, conservative treatment can be used when the neurological deficits improve during the early phase.^2^ Given that the abasia in case 1 did not improve 24 h after its onset, we performed hematoma removal and decompression by corpectomy from the front of C3–C5. In contrast, the symptoms in case 2 improved 24 h after onset; thus, conservative medical treatment was successfully used in achieving a positive outcome. Several reports showed that SSEH spontaneously resolved without surgery.^11^, ^12^ Therefore, conservative treatment is a logical option for elderly patients who present with neurological improvement.

## Conclusion

We reported two cases of SSEH in elderly Japanese men with dysstasia only and without back or neck pain. The findings of this study indicate that SSEH should be considered in the differential diagnosis of similar cases, especially in Japan.

## Disclosure

Approval of the research protocol: Yes.

Informed consent: Yes.

Registry and registration no. of the study/trial no.: None.

Animal studies no.: None.

Conflicts of interest: None.
